# A population-based matched cohort study of major congenital anomalies following COVID-19 vaccination and SARS-CoV-2 infection

**DOI:** 10.1038/s41467-022-35771-8

**Published:** 2023-01-06

**Authors:** Clara Calvert, Jade Carruthers, Cheryl Denny, Jack Donaghy, Lisa E. M. Hopcroft, Leanne Hopkins, Anna Goulding, Laura Lindsay, Terry McLaughlin, Emily Moore, Bob Taylor, Maria Loane, Helen Dolk, Joan Morris, Bonnie Auyeung, Krishnan Bhaskaran, Cheryl L. Gibbons, Srinivasa Vittal Katikireddi, Maureen O’Leary, David McAllister, Ting Shi, Colin R. Simpson, Chris Robertson, Aziz Sheikh, Sarah J. Stock, Rachael Wood

**Affiliations:** 1 Usher Institute, University of Edinburgh, Edinburgh, UK; 2 Public Health Scotland, Glasgow, Scotland; 3 Bennett Institute for Applied Data Science, Nuffield Department of Primary Care Health Sciences, University of Oxford, Oxford, UK; 4 Gloucestershire Hospitals NHS Foundation Trust, Gloucestershire, UK; 5 Institute of Nursing and Health Research, Ulster University, Newtownabbey, UK; 6 Population Health Research Institute, St George’s, University of London, London, UK; 7 School of Philosophy, Psychology and Language Sciences, University of Edinburgh, Edinburgh, UK; 8 Department of Non-Communicable Diseases Epidemiology, London School of Hygiene and Tropical Medicine, London, UK; 9 School of Health & Wellbeing, University of Glasgow, Glasgow, UK; 10 School of Health, Wellington Faculty of Health, Victoria University of Wellington, Wellington, New Zealand; 11 Department of Mathematics and Statistics, University of Strathclyde, Glasgow, UK

**Keywords:** Epidemiology, Respiratory tract diseases, Vaccines, SARS-CoV-2

## Abstract

Evidence on associations between COVID-19 vaccination or SARS-CoV-2 infection and the risk of congenital anomalies is limited. Here we report a national, population-based, matched cohort study using linked electronic health records from Scotland (May 2020-April 2022) to estimate the association between COVID-19 vaccination and, separately, SARS-CoV-2 infection between six weeks pre-conception and 19 weeks and six days gestation and the risk of [1] any major congenital anomaly and [2] any non-genetic major congenital anomaly. Mothers vaccinated in this pregnancy exposure period mostly received an mRNA vaccine (73.7% Pfizer-BioNTech BNT162b2 and 7.9% Moderna mRNA-1273). Of the 6731 babies whose mothers were vaccinated in the pregnancy exposure period, 153 had any anomaly and 120 had a non-genetic anomaly. Primary analyses find no association between any vaccination and any anomaly (adjusted Odds Ratio [aOR] = 1.01, 95% Confidence Interval [CI] = 0.83-1.24) or non-genetic anomalies (aOR = 1.00, 95% CI = 0.81-1.22). Primary analyses also find no association between SARS-CoV-2 infection and any anomaly (aOR = 1.02, 95% CI = 0.66-1.60) or non-genetic anomalies (aOR = 0.94, 95% CI = 0.57-1.54). Findings are robust to sensitivity analyses. These data provide reassurance on the safety of vaccination, in particular mRNA vaccines, just before or in early pregnancy.

## Abstract (web summary)

The risks of major congenital anomalies associated with SARS-CoV-2 vaccination in early pregnancy are not well understood. Here, the authors conduct a population-based cohort study using electronic health records from Scotland and find no evidence of an association, supporting vaccine safety in pregnancy.

## Introduction

SARS-CoV-2 infection in pregnancy is associated with severe COVID-19 symptoms, and adverse maternal and perinatal outcomes^1,2^, yet low levels of COVID-19 vaccination uptake in pregnant women persist in many settings^3–5^. Among the barriers affecting COVID-19 vaccine uptake in pregnancy are concerns around vaccine safety^6,7^. There is evidence of the safety of COVID-19 vaccination in pregnancy with respect to perinatal outcomes such as stillbirth and early pregnancy outcomes such as miscarriage^8,9^. However, there is very limited evidence on associations between either COVID-19 vaccination or SARS-CoV-2 infection in early pregnancy and the risk of congenital anomalies.

The few studies that have been published to date on the association between COVID-19 vaccination and the risk of congenital anomalies have found no evidence of an association^10–15^, but these all had important methodological weaknesses. Only two of these studies, for example, restricted the exposure to vaccination in the first trimester^11,14^ when the baby’s organs develop and hence the key risk period for teratogen exposure^16^. Furthermore, none of the studies comprehensively captured congenital anomalies, either only documenting anomalies identified on ultrasound or only looking at live births (+/− stillbirths) thus excluding terminations of pregnancy for anomalies, which are known to account for a large number of all major congenital anomalies in Europe^17^. The evidence on any association between maternal SARS-CoV-2 infection and offspring risk of congenital anomalies is also very sparse. The few published studies have shown no evidence of an association^18–20^, although they have similar methodological limitations to those looking at the impact of COVID-19 vaccination. By contrast, certain other viral infections in early pregnancy (e.g., rubella and Zika) are well-recognised causes of specific anomaly syndromes^17,21^. Fever associated with infections, and medicines used to treat infections, could also be associated with anomaly risk^17^.

Maximizing COVID-19 vaccine uptake among women that are pregnant or planning to become pregnant requires that they and their healthcare providers have access to key information on the safety of these vaccines, as well as the potential harms from SARS-CoV-2 infection, to inform their decision-making. Given the paucity of high-quality data on the risk of congenital anomalies with COVID-19 vaccine and SARS-CoV-2 infection, we conducted a national, population-based, matched cohort study using data for all residents in Scotland from the COVID-19 in Pregnancy in Scotland (COPS) cohort^22,23^. We estimated the association between any COVID-19 vaccination and, separately, a confirmed SARS-CoV-2 infection between six weeks pre-conception and 19 weeks and six days (19 + 6) gestation and the risk of any major congenital anomaly (hereafter “any anomaly”) and the risk of any non-genetic major congenital anomaly (i.e., an anomaly with no known genetic basis, hereafter “non-genetic anomaly”).

## Results

### Association between COVID-19 vaccination and major congenital anomalies: primary analyses

**Fig. 1:**
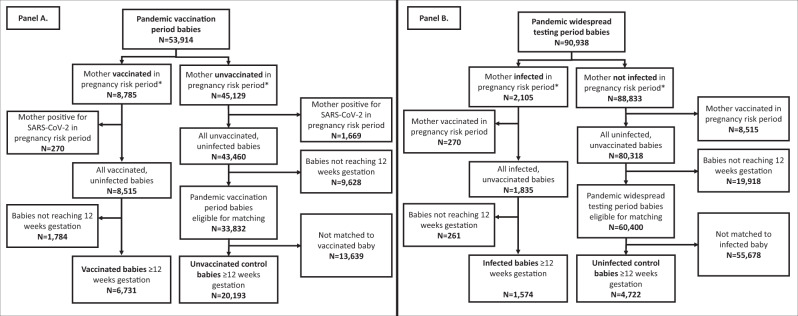
Selection of babies for the study. **A** shows the selection of babies for the analysis of association between COVID-19 vaccination and risk of major congenital anomalies. **B** shows the selection of babies for the analysis of the association between SARS-CoV-2 infection and risk of major congenital anomalies *Exposure needs to be between 6 weeks preconception and up to the earliest of: (1) end of pregnancy or (2) 19 weeks 6 days gestation.

The study dataset included 581,370 fetuses/babies (hereafter ‘babies’) resulting from all clinically recognized pregnancies in Scotland ending at any gestation and in any outcome (i.e., miscarriage, termination of pregnancy, stillbirth or live birth) from 2015 onwards. For the COVID-19 vaccination analyses, we identified 53,914 babies in the study vaccine exposure period (less than 19 + 6 gestation at, or conceived subsequent to, the start of the COVID-19 vaccination program on December 8, 2020) who had adequate follow-up time (Fig. 1). Of these 53,914 babies, 8785 had mothers who were vaccinated between six weeks preconception and 19 + 6 gestation (or the end of pregnancy if earlier). We excluded 270 babies where the mother also had SARS-CoV-2 infection in this pregnancy exposure period and 1784 babies where the pregnancy ended before 12 weeks gestation (as anomalies were not ascertained in early miscarriages or in the majority of early terminations of pregnancy), leaving a total of 6731 babies in our vaccinated cohort. These were matched (1:3) to 20,193 unvaccinated control babies (whose mothers were not vaccinated or infected in the relevant exposure period, and from pregnancies reaching at least 12 weeks gestation) on maternal age at conception and ensuring that the controls had reached at least the gestational week at which the mother was vaccinated.

Compared with matched babies of unvaccinated mothers, babies of vaccinated mothers were more likely to be born to mothers who were from the least deprived areas, did not smoke, and who had pre-existing medical conditions (Table 1). Of the 6731 babies of vaccinated mothers, 153 (22.7 per 1000 babies) had any anomaly identified. In 120 babies (17.8 per 1000), their anomaly had no known genetic basis. Of the 20,193 babies of unvaccinated mothers, 467 (23.1 per 1000) had any anomaly, and 375 (18.6 per 1000) had a non-genetic anomaly. The distribution of types of anomalies seen in the vaccinated and control cohorts were similar (Table 2).

**Table 1:** Key sociodemographic and clinical characteristics of vaccinated and control groups

	Vaccinated cohort	Unvaccinated controls
Number of pregnancies reaching ≥ 12 weeks gestation	6623	19,981
Total babies resulting from these pregnancies [live births]	6731 [6445]	20,193 [19,365]
Median maternal age (min-max) [standard deviation]	32 (16–46) [5.1]	32 (16–47) [4.9]
Maternal deprivation (SIMD quintile)*		
1 (most deprived)	1119 (16.6%)	4340 (21.5%)
2	1181 (17.5%)	3895 (19.3%)
3	1269 (18.9%)	3663 (18.1%)
4	1580 (23.5%)	4467 (22.1%)
5 (least deprived)	1582 (23.5%)	3811 (18.9%)
Unknown	0	17 (0.1%)
**Maternal ethnicity**		
White	6023 (89.5%)	16,949 (83.9%)
South Asian	243 (3.6%)	735 (3.6%)
Black/Caribbean /African	85 (1.3%)	435 (2.2%)
Other/mixed ethnicity	229 (3.4%)	845 (4.2%)
Unknown	151 (2.2%)	1229 (6.1%)
**Maternal urban/rural status**		
Large urban areas	2474 (36.8%)	7498 (37.1%)
Other urban areas	2240 (33.3%)	7082 (35.1%)
Accessible small towns	563 (8.4%)	1549 (7.7%)
Remote small towns	226 (3.4%)	585 (2.9%)
Accessible rural areas	818 (12.2%)	2349 (11.6%)
Remote rural areas	343 (5.1%)	918 (4.5%)
Unknown	67 (1.0%)	212 (1.0%)
Maternal clinical vulnerability**		
Not vulnerable	4803 (71.4%)	14,871 (73.6%)
Vulnerable	1822 (27.1%)	5156 (25.5%)
Extremely vulnerable	106 (1.6%)	166 (0.8%)
**Maternal diabetes**		
No - assumed & confirmed	6133 (91.1%)	18,536 (91.8%)
Pre-existing diabetes	114 (1.7%)	155 (0.8%)
Gestational Diabetes/onset unknown	484 (7.2%)	1502 (7.4%)
**Maternal smoking status**		
Non-smoker	4885 (72.6%)	13,857 (68.6%)
Ex-smoker	1292 (19.2%)	4033 (20.0%)
Smoker	523 (7.8%)	2227 (11.0%)
Unknown	31 (0.5%)	76 (0.4%)
**Maternal body mass index**		
Underweight	97 (1.4%)	406 (2.0%)
Healthy weight	2354 (35.0%)	7273 (36.0%)
Overweight	1978 (29.4%)	6207 (30.7%)
Obese/severely obese	2056 (30.5%)	5609 (27.8%)
Unknown	246 (3.7%)	698 (3.5%)
**Baby from singleton or multiple pregnancy**		
Singleton	6517 (96.8%)	19,579 (97.0%)
Multiple	214 (3.2%)	614 (3.0%)

*Maternal area-level deprivation was based on postcode of residence and derived according to the standard measure used by the Scottish Government (Scottish Index of Multiple Deprivation [SIMD]).

**Women were classified as clinically extremely vulnerable if they were identified on the national highest risk/shielding list maintained by Public Health Scotland ^46^ and, of those not extremely vulnerable, were classified as clinically vulnerable if they were in any Q-COVID risk group ^47^ (excluding diabetes) or had hypertension.

**Table 2:** Descriptive information on vaccination (vaccinated group only) and on the risk and types of major congenital anomalies in the vaccinated and controls groups

	Vaccinated cohort	Unvaccinated controls
**Exposure (vaccination)**		
Gestation at first vaccination within exposure period*		
Up to six weeks preconception	2045 (30.4%)	—
2 + 0-9 + 6 weeks	1925 (28.6%)	—
10 + 0-13 + 6 weeks	893 (13.3%)	—
14 + 0-19 + 6 weeks	1868 (27.8%)	—
**Number of vaccinations within exposure period**		
1	4871 (72.4%)	—
2+	1860 (27.6%)	—
**Dose number at first vaccination within exposure period**		
Dose 1	5516 (81.9%)	—
Dose 2	1204 (17.9%)	—
Dose 3	11 (0.2%)	—
**Type of vaccination within exposure period**		
BNT162b2	4963 (73.7%)	—
mRNA-1273	535 (7.9%)	—
ChAdOx1-S/nCoV-19	1222 (18.2%)	—
1+ different type	11 (0.2%)	—
**Outcome (major congenital anomaly)**		
Total *N* babies with any anomaly	153	467
Total *N* live births with any anomaly	110	335
Total live birth prevalence of any anomaly (/1000 live births)	17.1	17.3
Total *N* babies with non-genetic anomaly	120	375
Total *N* live births with non-genetic anomaly	94	295
Total live birth prevalence of non-genetic anomaly (/1000 live births)	14.6	15.2
***N*** **(%) babies with the following types of anomaly [*N*** **with non-genetic anomaly]**		
Nervous system	14 (9.2%) [13]	43 (9.2%) [41]
Eye	2 (1.3%) [2]	1 (0.2%) [0]
Ear, face and neck	2 (1.3%) [2]	2 (0.4%) [2]
Congenital heart defects	41 (26.8%) [35]	105 (22.5%) [84]
Respiratory	1 (0.7%) [1]	5 (1.1%) [5]
Oro-facial clefts	7 (4.6%) [3]	16 (3.4%) [13]
Digestive system	13 (8.5%) [12]	45 (9.6%) [38]
Abdominal wall defects	3 (2.0%) [3]	6 (1.3%) [6]
Urinary	11 (7.2%) [7]	31 (6.6%) [30]
Genital	15 (9.8%) [14]	43 (9.2%) [41]
Limb defect	23 (15.0%) [22]	75 (16.1%) [73]
Other anomalies/syndromes**	18 (11.8%) [9]	30 (6.4%) [18]
Chromosomal	24 (15.7%) [0]	80 (17.1%) [0]

*Between 6 weeks preconception and up to the earliest of: (1) end of pregnancy or (2) 19 weeks 6 days gestation.

**The “Other anomalies/syndromes group” includes a disparate range of conditions including genetic syndromes and microdeletions, skeletal dysplasias, and recognised teratogenic syndromes and associations. No unusual pattern in the distribution of these other anomalies was seen in the vaccinated group.

There was no evidence for an association between COVID-19 vaccination and any anomaly in analyses that only included matching factors (maternal age at conception and gestational week at matching) in the model (Odds Ratio [OR] = 0.98, 95% Confidence Interval [CI] 0.82–1.18) or which adjusted for all socio-demographic and clinical covariates (adjusted OR [aOR] = 1.01, 95% CI 0.83–1.24) (Table 3). Similarly, there was no evidence for an association between vaccination and non-genetic anomalies when accounting for the matching factors (OR = 0.96, 95% CI 0.78–1.18) or when additionally adjusting for the socio-demographic and clinical covariates (aOR = 1.00, 95% CI 0.81–1.22) (Table 3).

**Table 3:** Odds ratios for the association between COVID-19 vaccination and major congenital anomalies among babies reaching at least 12 weeks gestation, calculated using conditional logistic regression models

	*N* pregnancies	*N* total babies	*N* babies with any major congenital anomaly	Total prevalence (/1000 total babies)	OR (95% CI)*	*p* value*	Adjusted OR (95% CI)**	*p* value**
**Any major congenital anomaly**								
Unvaccinated	19,981	20,193	467	23.1	1		1	
Vaccinated	6623	6731	153	22.7	0.98 (0.82–1.18)	0.85	1.01 (0.83–1.24)	0.96
**Any non-genetic major congenital anomaly**								
Unvaccinated	19,981	20,193	375	18.6	1		1	
Vaccinated	6623	6731	120	17.8	0.96 (0.78–1.18)	0.69	1.00 (0.81–1.22)	0.98

*OR* odds ratio, *CI* confidence interval.

*Accounting for matching factors: maternal age at conception and gestational week at matching.

**Additionally adjusted for: maternal deprivation, maternal ethnicity, maternal urban rural status, maternal clinical vulnerability, maternal diabetes, maternal smoking status, maternal body mass index and whether the baby was from a singleton or multiple pregnancy.

### Association between COVID-19 vaccination and major congenital anomalies: sensitivity and subgroup analyses

Results were similar in analyses including babies from pregnancies of any duration (not just those reaching at least 12 weeks gestation) (Supplementary Table 1). When we restricted our vaccinated cohort to babies whose mothers were vaccinated in the key teratogenic risk window of conception (at 2 + 0 gestation) to 9 + 6 gestation inclusive, we again found no evidence of an association between vaccination and risk of anomalies: our adjusted OR for the association between vaccination and any anomaly was 0.76 (95% CI 0.52–1.10) and non-genetic anomalies was 0.66 (95% CI 0.43–1.02) (Supplementary Table 1).

The mothers vaccinated in the pregnancy exposure period mostly received an mRNA vaccine (73.7% Pfizer-BioNTech BNT162b2 and 7.9% Moderna mRNA-1273), 18.2% were exposed to the viral vector Oxford-AstraZeneca ChAdOx1-s/nCoV-19 vaccine, and 0.2% were exposed to more than one vaccine type (Table 2). Women receiving mRNA and viral vector vaccines had substantially different characteristics, reflecting the roll out of the vaccination programme in Scotland (Supplementary Table 2; Supplementary Fig. 1). Compared to women receiving an mRNA vaccine, and to their matched controls, women receiving the viral vector ChAdOx1-s/nCoV-19 vaccine were more likely to be from deprived areas, to smoke, to be overweight or obese, and much more likely to have pre-existing medical conditions. After accounting for the matching factors and adjusting for covariates, there was no evidence of an association between mRNA vaccination and any anomaly (aOR = 0.92, 95% CI 0.73–1.14) or non-genetic anomalies (aOR = 0.85, 95% CI 0.66–1.10) (Supplementary Table 3). After accounting for the matching factors and adjusting for covariates, the adjusted OR estimating the association between ChAdOx1-s/nCoV-19 vaccination and any anomaly was 1.35 (95% CI 0.90–2.03), and non-genetic anomalies was 1.56 (95% CI 0.97–2.51). Point estimates for these associations were therefore raised, but with wide confidence intervals spanning one (Supplementary Table 3).

### Association between SARS-CoV-2 infection and major congenital anomalies: primary analyses

We identified 90,938 babies whose mothers could have had confirmed infection with SARS-CoV-2 in the relevant exposure period (less than 19 + 6 gestation at, or conceived subsequent to, the introduction of widespread community testing for SARS-CoV-2 on May 18, 2020) and who had adequate follow up time available (Fig. 1). There were 1574 babies from pregnancies that reached at least 12 weeks gestation whose mother had confirmed SARS-CoV-2 infection between six weeks preconception and 19 + 6 gestation (but did not have COVID-19 vaccination in this pregnancy exposure period) and were included in the infected cohort. These were matched (1:3) to 4722 control babies who were not exposed to SARS-CoV-2 infection (or COVID-19 vaccination) in the pregnancy exposure period on maternal age at conception, gestational week of infection and, given the longer study period of widespread testing compared with the vaccination period, additionally for season of conception.

Compared with matched babies of uninfected mothers, the babies exposed to confirmed SARS-CoV-2 infection were more likely to be born to mothers who were from the most deprived areas and from urban areas (Table 4). Of the 1574 babies exposed to confirmed SARS-CoV-2, 32 (20.3 per 1000 babies) had any anomaly and 26 (16.5 per 1000) had a non-genetic anomaly (Table 5). Of the 4722 babies not exposed to confirmed SARS-CoV-2 infection, 85 (18.0 per 1000) had any anomaly and 71 (15.0 per 1000) had a non-genetic anomaly.

**Table 4:** Key sociodemographic and clinical characteristics of infected and control groups

	Infected cohort	Uninfected controls
Number of pregnancies reaching ≥12 weeks gestation	1551	4719
Total babies resulting from these pregnancies [live births]	1574 [1510]	4722 [4554]
Median maternal age (min-max) [standard deviation]	29 (14–43) [5.3]	29 (15–44) [5.2]
Maternal deprivation (SIMD quintile)*		
1 (most deprived)	450 (28.6%)	1162 (24.6%)
2	393 (25.0%)	971 (20.6%)
3	261 (16.6%)	830 (17.6%)
4	261 (16.6%)	995 (21.1%)
5 (least deprived)	209 (13.3%)	760 (16.1%)
Unknown	0	4 (0.1%)
**Maternal ethnicity**		
White	1415 (89.9%)	3944 (83.5%)
South Asian	69 (4.4%)	174 (3.7%)
Black/Caribbean /African	21 (1.3%)	74 (1.6%)
Other/mixed ethnicity	47 (3.0%)	167 (3.5%)
Unknown	22 (1.4%)	363 (7.7%)
**Maternal urban/rural status**		
Large urban areas	665 (42.2%)	1670 (35.4%)
Other urban areas	616 (39.1%)	1742 (36.9%)
Accessible small towns	103 (6.5%)	402 (8.5%)
Remote small towns	31 (2.0%)	142 (3.0%)
Accessible rural areas	114 (7.2%)	523 (11.1%)
Remote rural areas	38 (2.4%)	195 (4.1%)
Unknown	7 (0.4%)	48 (1.0%)
Maternal clinical vulnerability**		
Not vulnerable	1141 (72.5%)	3480 (73.7%)
Vulnerable	425 (27%)	1206 (25.5%)
Extremely vulnerable	8 (0.5%)	36 (0.8%)
**Maternal diabetes**		
No - assumed & confirmed	1444 (91.7%)	4375 (92.7%)
Pre-existing diabetes	12 (0.8%)	51 (1.1%)
Gestational Diabetes/onset unknown	118 (7.5%)	296 (6.3%)
**Maternal smoking status**		
Non-smoker	1121 (71.2%)	3112 (65.9%)
Ex-smoker	298 (18.9%)	970 (20.5%)
Smoker	148 (9.4%)	615 (13%)
Unknown	7 (0.4%)	25 (0.5%)
**Maternal body mass index**		
Underweight	20 (1.3%)	137 (2.9%)
Healthy weight	557 (35.4%)	1769 (37.5%)
Overweight	505 (32.1%)	1374 (29.1%)
Obese/severely obese	452 (28.7%)	1295 (27.4%)
Unknown	40 (2.5%)	147 (3.1%)
**Baby from singleton or multiple pregnancy**		
Singleton	1529 (97.1%)	4614 (97.7%)
Multiple	45 (2.9%)	108 (2.3%)

*Maternal area-level deprivation was based on postcode of residence and derived according to the standard measure used by the Scottish Government (Scottish Index of Multiple Deprivation [SIMD]).

**Women were classified as clinically extremely vulnerable if they were identified on the national highest risk/shielding list maintained by Public Health Scotland^46^ and, of those not extremely vulnerable, were classified as clinically vulnerable if they were in any Q-COVID risk group^47^ (excluding diabetes) or had hypertension

**Table 5:** Descriptive information on infection exposure (infected group only) and on the risk and types of major congenital anomalies in the infected and controls groups

	Infected cohort	Uninfected controls
**Exposure (SARS-CoV-2 infection)**		
Gestation at first infection within exposure period*		
Up to six weeks preconception	257 (16.3%)	—
2 + 0-9 + 6 weeks	481 (30.6%)	—
10 + 0-13 + 6 weeks	265 (16.8%)	—
14 + 0-19 + 6 weeks	571 (36.3%)	—
**Number of infections within exposure period**		
1	1570 (99.7%)	—
2+	4 (0.3%)	—
**Outcome (major congenital anomaly)**		
Total *N* babies with any anomaly	32	85
Total *N* live births with any anomaly	24	63
Total live birth prevalence of any anomaly (/1000 live births)	15.9	13.8
Total *N* babies with non-genetic anomaly	26	71
Total *N* live births with non-genetic anomaly	22	58
Total live birth prevalence of non-genetic anomaly (/1000 live births)	14.6	12.7
***N*** **(%) babies with the following types of anomaly [***N* **with non-genetic anomaly]**		
Nervous system	2 (6.2%) [2]	7 (8.2%) [7]
Eye	0	0
Ear, face and neck	0	1 (1.2%) [1]
Congenital heart defects	8 (25%) [7]	26 (30.6%) [22]
Respiratory	0	0
Oro-facial clefts	2 (6.2%) [1]	3 (3.5%) [3]
Digestive system	4 (12.5%) [4]	7 (8.2%) [6]
Abdominal wall defects	1 (3.1%) [1]	2 (2.4%) [2]
Urinary	2 (6.2%) [2]	6 (7.1%) [5]
Genital	1 (3.1%) [1]	6 (7.1%) [6]
Limb defect	9 (28.1%) [9]	18 (21.2%) [17]
Other anomalies/syndromes**	4 (12.5%) [3]	2 (2.4%) [0]
Chromosomal	5 (15.6%) [0]	12 (14.1%) [0]

*Between 6 weeks preconception and up to the earliest of: (1) end of pregnancy or (2) 19 weeks 6 days gestation

**The Other anomalies/syndromes group includes a disparate range of conditions including genetic syndromes and microdeletions, skeletal dysplasias, and recognised teratogenic syndromes and associations. No unusual pattern in the distribution of these other anomalies was seen in the infected group

There was no evidence for an association between SARS-CoV-2 infection and any anomaly in analyses including matching factors (OR = 1.13, 95% CI 0.75–1.70) or when additionally adjusting for all socio-demographic and clinical covariates (aOR = 1.02, 95% CI 0.66–1.60) (Table 6). Similarly, there was no evidence for an association between SARS-CoV-2 infection and non-genetic anomalies when accounting for the matching factors (OR = 1.10, 95% CI 0.70–1.73) or when also adjusting for the socio-demographic and clinical covariates (aOR = 0.94, 95% CI 0.57–1.54) (Table 6).

**Table 6:** Odds ratios for the association between SARS-CoV-2 infection and major congenital anomalies among babies reaching at least 12 weeks gestation, calculated using conditional logistic regression models

	*N* pregnancies	*N* total babies	*N* babies with any major congenital anomaly	Total prevalence (/1000 total babies)	OR (95% CI)*	*p* value*	Adjusted OR (95% CI)**	*p* value**
**Any major congenital anomaly**								
Uninfected	4719	4722	85	18.0	1		1	
Infected	1551	1574	32	20.3	1.13 (0.75–1.70)	0.56	1.02 (0.66–1.60)	0.92
**Any non-genetic major congenital anomaly**								
Uninfected	4719	4722	71	15.0	1		1	
Infected	1551	1574	26	16.5	1.10 (0.70–1.73)	0.68	0.94 (0.57–1.54)	0.80

*OR* odds ratio, *CI* confidence interval

*Accounting for matching factors: maternal age at conception, season of conception and gestational week at matching

**Additionally adjusted for: maternal deprivation, maternal ethnicity, maternal urban rural status, maternal clinical vulnerability, maternal diabetes, maternal smoking status, maternal body mass index and whether the baby was from a singleton or multiple pregnancy

### Association between SARS-CoV-2 infection and major congenital anomalies: sensitivity analyses

Results were similar in sensitivity analysis including babies from pregnancies of any duration (not just those reaching at least 12 weeks gestation) (Supplementary Table 4). When we restricted our infected cohort to babies whose mothers were infected between conception and 9 + 6 gestation inclusive, our adjusted OR for the association between SARS-CoV-2 infection and any anomaly was 1.14 (95% CI 0.57–2.29) and non-genetic anomalies was 1.05 (95% CI 0.47–2.34) (Supplementary Table 4).

## Discussion

In this national, population-based, matched cohort study in Scotland, we find no evidence in our primary analyses that maternal exposure to any COVID-19 vaccination between six weeks pre-conception and 19 + 6 weeks gestation was associated with an increased risk of either any anomaly or non-genetic anomalies among babies that reached at least 12 weeks gestation. Similarly, there was no evidence of an association between maternal exposure to confirmed SARS-CoV-2 infection in this period and any anomaly or non-genetic anomalies. These results were robust in sensitivity analyses including all babies (not just those reaching 12 weeks gestation).

There remained no evidence for an elevated risk of any anomaly when we narrowed the exposure window to the key teratogenic risk period of between conception and 9 + 6 weeks gestation. The association between maternal infection in this narrower risk window and non-genetic anomalies was essentially unchanged. By contrast, maternal vaccination in this narrower risk window was associated with a slightly lower risk of non-genetic anomalies. Confidence intervals were wide due to relatively small numbers of babies exposed to maternal vaccination in the narrow window. It is plausible, however, that this reflects a particularly strong “healthy vaccinee bias”^24^ with women who were motivated to get COVID-19 vaccination in this early stage of pregnancy different in their health and/or health-related behaviours compared to their unvaccinated controls. The healthy vaccinee bias may also affect our other vaccination analyses, although we have adjusted for a number of maternal characteristics that should account for this to a certain extent (e.g., maternal deprivation, BMI and smoking status).

This lack of association between vaccination in pregnancy and the risk of anomalies in babies aligns with existing evidence from a vaccine adverse event reporting system in the US^10,25^, facility-based studies comparing outcomes in vaccinated and unvaccinated pregnancies in the UK and the US^13,14^, as well as self-reported data on vaccination status and fetal anomaly identified at ultrasound from an online survey^12^. The most robust evidence published previously is a population-based cohort study from Israel conducted by Goldshtein and colleagues^11^. They found no evidence that singleton, live births to women who were vaccinated in the first trimester had a higher risk of congenital anomalies compared with those not exposed to vaccination in pregnancy (Risk Ratio = 0.69, 95% CI = 0.44–1.04).

All the studies published to-date either only included pregnancies exposed to mRNA vaccinations or looked at all vaccines overall and did not stratify by vaccine type. We conducted pre-planned subgroup analysis by the type of vaccination (mRNA or viral-vector). The majority of mothers vaccinated in the pregnancy exposure period received an mRNA vaccine (81.6%), and we found no evidence for an association between mRNA vaccination and either any anomaly or non-genetic anomalies. However, while the confidence intervals are wide, we cannot rule out a modest increase in the risk of any anomaly (aOR = 1.35, 95% CI 0.90–2.03) or non-genetic anomalies (aOR = 1.56, 95% CI 0.97–2.51) with the viral-vector vaccination (Oxford-AstraZeneca ChAdOx1-s/nCoV-19). We urge caution in the interpretation of these results for two main reasons. Firstly, there was a relatively small sample size of babies whose mother received ChAdOx1-s/nCoV-19 in the pregnancy exposure period and consequently we have wide confidence intervals. Secondly, there is potential for residual confounding, with mothers of babies exposed to this vaccine having quite different characteristics compared to both their unvaccinated controls and those of babies exposed to the mRNA vaccine. These differences reflect the roll out of the vaccination programme in Scotland. The Pfizer/BioNTech BNT162b2 vaccine was used from the start of the vaccination programme on December 8, 2020. The Oxford-AstraZeneca ChAdOx1-s/nCoV-19 vaccine was introduced from January 4, 2021, and the Moderna mRNA-1273 vaccine from April 7, 2021. In the early months of the vaccine programme, BNT162b2 was mainly offered to health and social care workers at high risk of occupational exposure to SARS-CoV-2 (as these groups could be vaccinated in centralised facilities that could accommodate the ultra-cold storage requirements for this vaccine), and ChAdOx1-s/nCoV-19 was offered to individuals at risk of severe COVID-19 disease due to pre-existing medical conditions (often vaccinated in local General Practitioner surgeries). In mid-April 2021, prior to vaccination being rolled out to all younger adults (without pre-existing conditions) including pregnant women, the UK’s Joint Committee on Vaccination and Immunisation (JCVI) recommended that pregnant women commencing a course of vaccination from that point on should be offered an mRNA vaccine rather than ChAdOx1-s/nCoV-19. This reflected accumulating evidence on the safety of mRNA vaccines in pregnancy following their extensive use in the US rather than any specific concern about the use of viral vector vaccines in pregnancy^26^. The pregnant women in our study receiving ChAdOx1-s/nCoV-19 will therefore mainly be those identified as clinically vulnerable due to pre-existing medical conditions: this group will be at increased risk of a range of adverse pregnancy outcomes, including anomalies. It is therefore reassuring that, whilst the risk of anomalies was slightly elevated in babies from this group, the distribution of types of anomalies seen was similar to that in controls. The distribution was also in line with expectations based on whole population data from Scotland in 2019 which showed that the most common types of major congenital anomalies were congenital heart defects, nervous system, limb and/or chromosomal congenital anomalies, with ear, face, neck, eye, respiratory and abdominal wall defects the rarest anomalies^27^. Nevertheless, data from settings with a different profile of women receiving ChAdOx1-s/nCoV-19 would be beneficial in confirming the safety profile of this vaccine.

Our study also adds to a growing body of epidemiological evidence that maternal SARS-CoV-2 infection is not associated with the risk of congenital anomalies^18–20^. We recognise that we may have misclassified some mothers who actually had SARS-CoV-2 infection as uninfected if they did not have a positive SARS-CoV-2 reverse transcription (RT) polymerase chain reaction (PCR) test result. To minimise the number of missed infections, we restricted our analysis to the period when widespread community-based testing was available, which ensured access to testing for a wide range of people, for example, anyone with symptoms and for anyone being admitted to hospital^28,29^. Studies so far have suggested that transmission of SARS-CoV-2 across the placenta is rare^30,31^, which likely explains the lack of association. There has, however, been a case report of a severe eye anomaly linked to maternal SARS-CoV-2 infection at 5-6 weeks gestation^32^. We did not identify any eye anomalies in babies exposed to infection in early pregnancy (or their controls), but this highlights the importance of continued monitoring of congenital anomalies following SARS-CoV-2 infection.

The strengths of this study include that it was population-based and, compared to most previous studies, had a large sample size. Unlike many previous studies we restricted our exposure period to the relevant early pregnancy period, and we examined both a broader exposure window, between six weeks preconception and 19 + 6 weeks gestation, and a narrower window reflecting the key teratogenic risk period of between conception and 9 + 6 weeks. Critically, we included babies regardless of pregnancy outcome in our analyses, hence babies with an anomaly will be included whether the pregnancy ends in a termination or a live or stillbirth. The presence or absence of an anomaly is usually unknown in babies where the pregnancy ends in an early miscarriage or social termination. We therefore restricted our primary analyses to only include babies from pregnancies reaching at least 12 weeks gestation but undertook sensitivity analyses including babies from pregnancies of all duration.

There are, however, also some important limitations. Firstly, there were only small numbers of specific types of congenital anomalies preventing any subgroup analyses by type of anomaly. We have, however, presented descriptive data for more specific types of congenital anomalies and these were in line with the numbers expected based on previously published data from Scotland^27^. Secondly, while we used robust methods for the ascertainment and classification of anomalies based on international (EUROCAT) standards^33^, we are unlikely to have identified all congenital anomalies in our study population. Specifically, we will have under-ascertained anomalies among live births (particularly those that are not evident shortly after birth) as we curtailed follow-up for congenital anomalies to 28 days, rather than the usual 12 months, after birth to allow timely analyses. We also were not able to integrate data on the measurement of the occipito-frontal circumference at birth for comprehensive assessment of microcephaly, originally proposed in our study protocol as an outcome of interest, as these data were too incomplete. In primary analyses, we will have additionally excluded a small number of babies with anomalies that were subject to termination of pregnancy before 12 weeks gestation, although our sensitivity analyses retaining all babies regardless of gestation indicated that this had negligible impact. More speculatively, it is possible that pandemic-related changes in maternity and neonatal care, and associated national data quality, may have influenced the ascertainment of anomalies, however this would be unlikely to impact our exposed and controls groups differentially. Finally, and common to all observational studies, is the challenge of ensuring adequate adjustment for potential confounders. We included a number of potentially important confounders, including clinical vulnerability and smoking, but found that adjustment had little impact on the OR. We did not have data on some potential confounders, for example, parity and other major teratogenic exposures such as use of teratogenic medications or infections known to increase the risk of congenital anomalies (e.g., rubella), but this is unlikely to explain the absence of association that we observed in this study. We were also unable to match on pregnancy start date or season of conception when selecting unvaccinated controls due to the relatively small available pool of unvaccinated women who were the same maternal age and had the same pregnancy start date; additional exploratory analyses matching on season of conception and using fewer unvaccinated controls did not change our conclusions.

In summary, this study provides some of the most robust evidence to date that there is no association between COVID-19 vaccination or SARS-CoV-2 infection and the risk of major congenital anomalies. However, even using national, population-based data, we were not able to look at specific types of congenital anomalies, highlighting the importance of continued monitoring of major congenital anomalies and pooling of data from different settings to look at these rarer events. More generally, our study adds to a growing body of evidence on the safety of COVID-19 vaccination (in particular using mRNA vaccines) in pregnancy and provides important reassurance on safety of vaccination just before or in early pregnancy, although more data are required from other settings on viral vector vaccines. This supports current policy and clinical advice that vaccination can be given at any stage of pregnancy and to women planning to become pregnant^26,34,35^, and that vaccination remains the best way for women to protect themselves and their babies from the known risks of SARS-CoV-2 infection in late pregnancy.

## Methods

This population-based, matched cohort study was conducted according to a pre-specified study protocol and statistical analysis plan^36^, with our results reported and recorded according to the Strengthening the Reporting of Observational Studies in Epidemiology (STROBE) guidelines^37^.

### Ethical and governance approvals

The National Research Ethics Service Committee, South East Scotland 02 provided ethical approval (REC 12/SS/0201: SA 2). The Public Benefit and Privacy Panel for Health and Social Care (PBPP-HSC) provided information governance approval (2021-0116). Under the terms of our PBPP-HSC approval, patient consent was not required for use of routine health records for the COPS study.

### Study population

We used the COVID-19 in Pregnancy in Scotland (COPS) cohort as updated in mid-July, 2022. The COPS cohort includes information on all completed and ongoing pregnancies (and the resulting fetuses/babies - hereafter ‘babies’ for brevity) in Scotland from January 1, 2015 onwards (*N* = 581,370). Pregnancies ending at any gestation and in any outcome (miscarriage, termination of pregnancy, stillbirth, live birth) were included. Information on pregnancies, including estimated date of conception, pregnancy outcome, and gestational age at end of pregnancy, were extracted from: antenatal care (ANC) booking records; General Practitioner (GP) records; general acute hospital discharge records (Scottish Morbidity Record (SMR) 01); maternity hospital discharge records (SMR 02); statutory termination of pregnancy notifications (AAS); National Records of Scotland (NRS) statutory live birth and stillbirth registrations; and NHS Scotland live birth notifications. Further details on the creation of the COPS dataset have been published elsewhere^22,23^. In general, date of conception was estimated from the date and recorded gestation at the end of pregnancy (or date and recorded gestation at antenatal booking (i.e. the first contact a pregnant woman has with a midwife to plan ongoing care for the pregnancy and birth^38^)). Recorded gestation at the end of pregnancy is highly complete and accurate on national records relating to pregnancies ending in termination, late miscarriage, or delivery, but is usually missing for pregnancies ending in early miscarriage occurring before antenatal booking. Imputed gestation at the end of pregnancy was therefore used for any completed pregnancy with unknown gestation, with the imputed gestation based on pregnancy outcome, for example 10 + 0 weeks for miscarriages managed in non-maternity settings (and hence likely to be early losses).

For this study, we included babies with an estimated conception date before June 2, 2021. This ensures that all babies could be followed up to 40 weeks and six days (40 + 6) gestation by the end of February 2022, with sufficient time then allowed for return of national health records relating to care provided at the end of pregnancy or (for live births) during the subsequent 28-day neonatal period, and their incorporation into the COPS dataset update in mid-July 2022. A small number of completed pregnancies with an unknown pregnancy outcome were excluded from this analysis (*N* = 5738, 1.0%).

### Exposure and outcome information

National data on COVID-19 vaccination (including date and type of vaccination) and confirmed SARS-CoV-2 infections were incorporated into the COPS study cohort using unique identifiers to identify exposures of interest.

A modified version of the Scottish Linked Congenital Condition database (SLiCCD)^39^ was updated in July 2022 and linked to the COPS study cohort using unique identifiers to identify babies with major congenital anomalies. The SLiCCD is usually updated on an annual basis by Public Health Scotland (PHS) to enable surveillance of the occurrence of anomalies in the Scottish population^27^. Production of the SLiCCD involves linkage and analysis of a range of national health datasets held by PHS (maternity hospital discharge records (SMR 02); statutory termination of pregnancy notifications (AAS); NRS statutory stillbirth registrations; hospital neonatal care (Scottish Birth Record), general including paediatric acute discharge records (SMR 01), and NRS statutory death registration records for babies; and MBRRACE perinatal death enhanced surveillance records)^40^. Diagnostic codes on the source records are used to identify babies with a major structural or chromosomal anomaly or recognised syndrome meeting EUROCAT inclusion criteria^41^, specifically EUROCAT Guide 1.4 which was in place at the time this study started^33^. In line with EUROCAT guidance, babies are included in SLiCCD if the pregnancy ends in a termination of pregnancy at any gestation, a spontaneous pregnancy loss at 20 weeks gestation or over, or a live birth. Usually, SLiCCD includes live born babies diagnosed at any point up to their first birthday. To allow more timely analyses, the modified version of SLiCCD produced for this study included babies diagnosed at any point up to end of the 28-day neonatal period. In addition, MBRRACE perinatal death enhanced surveillance records were not used due to long data lags.

In a deviation from our study protocol, we did not assess whether there was any association between either COVID-19 vaccination or SARS-CoV-2 infection and the risk of microcephaly, as the data on the measurement of the occipito-frontal circumference at birth were too incomplete to support these analyses.

### COVID-19 vaccination and major congenital anomalies

#### Exposure

Our primary exposure was receipt of any COVID-19 vaccine from six weeks before conception to 19 + 6 weeks gestation or end of pregnancy if earlier (in line with guidance from The Brighton Collaboration Congenital Anomalies Working Group^16^). As noted by The Brighton Collaboration Congenital Anomalies Working Group, this exposure window allows for potential errors in assigning a date of conception, while focusing on exposure within the most plausible gestational period for development of congenital anomalies (which varies between different types of congenital anomalies)^16^. Babies were categorized as having been exposed to vaccination if their mother received any vaccine type available in Scotland (Oxford/AstraZeneca ChAdOx1-S/nCoV-19, Pfizer-BioNTech BNT162b2, or Moderna mRNA-1273), any dose (first, second or third) and any number of doses (one dose or two doses) in this pregnancy exposure period.

#### Outcomes

For our primary analyses, all fetuses/babies from pregnancies that reached at least 12 weeks gestation were categorized as having a major congenital anomaly (major structural or chromosomal anomaly or recognised syndrome meeting EUROCAT inclusion criteria^33^) or not. The group with no major anomaly identified will include a small number of babies (from miscarriages at 12–19 weeks gestation inclusive) where anomaly status is unknowable/unknown. Following SLiCCD usual practice, we allocated each anomaly present in an affected baby to the relevant EUROCAT anomaly group (nervous system anomalies, congenital heart defects, etc). One baby could therefore be allocated to multiple groups, and the number of anomalies allocated to each group will sum to more than the total number of affected babies. Finally, we identified the subset of babies who had no known genetic basis for their anomaly (babies with no anomaly allocated to the chromosomal; skeletal dysplasias; or genetic syndromes and microdeletions EUROCAT (sub)groups), as anomalies with a genetic basis are not caused by environmental exposures such as medicines/vaccines.

#### Matching to unvaccinated controls

We matched each vaccinated baby to three babies from the pandemic vaccination period, from pregnancies reaching at least 12 weeks gestation whose mothers were unvaccinated in the pregnancy exposure period. Matching was on the basis of maternal age at conception (+/− one year) and gestational week of first maternal vaccination in the exposed baby (i.e., the exposed baby could be matched with any baby from the eligible control cohort which had reached at least the gestational week of the exposed baby at vaccination; hence a baby whose mother was vaccinated at 14 weeks gestation would be matched to a baby from a pregnancy reaching at least 14 weeks gestation). Maternal age is particularly strongly associated with both the likelihood of being vaccinated and the risk of certain congenital anomalies. Gestational age at exposure is also associated with the risk of certain congenital anomalies (e.g. exposures after neural tube closure cannot be associated with neural tube defects). The number of potential controls was too small to allow additional matching by calendar time or season of conception for our primary analyses.

Given our focus on the safety of COVID-19 vaccination, all babies in whom the mother had SARS-CoV-2 infection between six weeks preconception and 19 + 6 weeks gestation (or the end of pregnancy if earlier) were excluded before the matching was conducted. As such, we were only able to capture the direct association between COVID-19 vaccination and congenital anomalies and not any impact of COVID-19 vaccination mediated through reducing the risk and severity of SARS-CoV-2 infection.

### SARS-CoV-2 infection and major congenital anomalies

We took a similar approach to prepare the COPS dataset to assess whether there was any association between confirmed SARS-CoV-2 infection between six weeks preconception and 19 + 6 weeks gestation (or end of pregnancy if earlier) and the risk of congenital anomalies. In line with national guidance in place over the study period^42^, confirmed infection was defined as a positive SARS-CoV-2 reverse transcription (RT) polymerase chain reaction (PCR) test result, and date of onset was taken as the date of the first positive test within the pregnancy exposure period. If a woman had multiple positive tests, these were considered separate infections if they were >90 days apart. To minimize the risk of misclassifying babies as unexposed to infection in the pregnancy exposure period due to lack of SARS-CoV-2 testing, we restricted the study population for this analysis to babies that had not yet reached 19 + 6 weeks gestation or were conceived after the date when widespread community testing became available in Scotland (May 18, 2020). Three babies not exposed to infection in the pregnancy exposure period were matched to each infected baby on maternal age at conception (+/− 1 year), gestational week of first maternal infection in the exposed baby, and season of conception. All babies in which the mother had COVID-19 vaccination between six weeks preconception and 19 + 6 weeks gestation (or the end of pregnancy if earlier) were excluded before the matching was conducted.

### Covariates

Data on potential sociodemographic and clinical confounders of the association between both COVID-19 vaccination and SARS-CoV-2 infection with congenital anomalies were available in the COPS data. Specifically, data were available on maternal area-level deprivation (based on population quintiles with “1” indicating the most deprived fifth of the Scottish population, and “5” the least deprived^43^), maternal ethnicity (grouped according to the Scottish decennial population census categories^44^), maternal rural urban status (categorized into six groups for descriptive analyses but as urban or rural for modelling^45^), maternal clinical vulnerability (not clinically vulnerable, clinically vulnerable (but excluding diabetes) or clinically extremely vulnerable), maternal diabetes (none, pre-existing diabetes, gestational diabetes), maternal smoking status (non-smoker, ex-smoker, smoker, unknown), maternal body mass index (BMI) (underweight, healthy weight, overweight, obese and severely obese, unknown) and whether the baby was from a singleton or multiple pregnancy. Maternal area-level deprivation was based on postcode of residence and derived according to the standard measure used by the Scottish Government (Scottish Index of Multiple Deprivation [SIMD]) which ranks areas by deprivation on the basis of seven domains: income, employment, education, health, access to services, crime and housing^43^. Women were classified as clinically extremely vulnerable if they were identified on the national highest risk/shielding list maintained by Public Health Scotland^46^ and, of those not extremely vulnerable, were classified as clinically vulnerable if they were in any Q-COVID risk group^47^ (excluding diabetes) or had hypertension according to cross-sectional GP/primary care data available from June 2020 and January 2021. Data on maternal diabetes, and BMI at antenatal booking, were extracted from delivery records where possible, then from GP records if available. Similarly, smoking status at antenatal booking was extracted from delivery records then GP records, but with one exception. If it was documented that a woman was a non-smoker at antenatal booking, but they were recorded as either a smoker or ex-smoker on a GP record, then we categorized the woman as an ex-smoker.

### Statistical analysis

Descriptive analyses were initially conducted looking at the distribution of our exposed and control cohorts by sociodemographic and clinical characteristics (including assessing levels of missing data), looking at the distribution and prevalence of congenital anomalies by cohort and, for our exposed groups only, looking at further descriptive information about the exposures. For the babies exposed to vaccination, for example, we looked at the number and type of vaccinations administered to the mother in the pregnancy exposure period.

To assess whether there was any evidence of an association between our exposures and any anomaly, we used conditional logistic regression to account firstly for only the matching factors (maternal age and gestational age at matching for the vaccination analysis, and additionally for season of conception for the infection analysis), and then adjusting for all the sociodemographic and clinical covariates. For the few covariates with missing data, we included “unknown” groups in our main models so these babies were not dropped from the analysis. The same approach was taken to look at the non-genetic anomalies outcome.

Analyses were conducted in R 3.6.1.

### Subgroup and sensitivity analyses

We conducted pre-specified subgroup analyses, stratifying our analysis of the association between vaccination and congenital anomalies by whether the mother received mRNA vaccination (i.e., Pfizer-BioNTech BNT162b2 or Moderna mRNA-1273) or a viral vector vaccination (i.e., Oxford-AstraZeneca ChAdOx1-s/nCoV-19). Babies where the mother received different vaccinations between six weeks conception and 19 + 6 weeks gestation, and their controls, were excluded from these subgroup analyses.

We conducted two sensitivity analyses for the analysis of the association between both vaccination or SARS-CoV-2 infection and congenital anomalies. Firstly, we assessed the impact of including babies from pregnancies of any duration (not just those reaching at least 12 weeks). Secondly, we restricted our pregnancy exposure period to the key teratogenic risk window from conception to 9 + 6 weeks gestation inclusive.

### Reporting summary

Further information on research design is available in the Nature Portfolio Reporting Summary linked to this article.

## Supplementary information

Supplementary Information

Peer Review File

Reporting Summary

## Data Availability

Aggregate data files on COVID-19 vaccinations and SARS-CoV-2 infections among pregnant women are available here: https://www.opendata.nhs.scot/organization/health_protection. Patient-level data underlying this article cannot be shared publicly due to data protection and confidentiality requirements. Public Health Scotland is the data holder for the data used in this study. Data can be made available to approved researchers for analysis after securing relevant permissions from the data holders via the Public Benefit and Privacy Panel. Enquiries regarding data availability should be directed to phs.edris@phs.scot.

## Code availability

Metadata and code are available on GitHub at https://github.com/Public-Health-Scotland/COPS-public^48^.
