# Supplementary material for: A population-based matched cohort study of major congenital anomalies following COVID-19 vaccination and SARS-CoV-2 infection
Source: Nat Commun. 2023 Jan 6;14:107. doi: 10.1038/s41467-022-35771-8 (PMC9821346; doi:10.1038/s41467-022-35771-8)
Supplement: Supplementary file 1 — Supplementary Information [file 41467_2022_35771_MOESM1_ESM.pdf]

**Supplementary Information**

## Supplementary Figures

*Supplementary Figure 1: Number of babies exposed to COVID-19 vaccination between six weeks preconception and up to 19 weeks and six days gestation by the calendar time, dose number and type of vaccine*

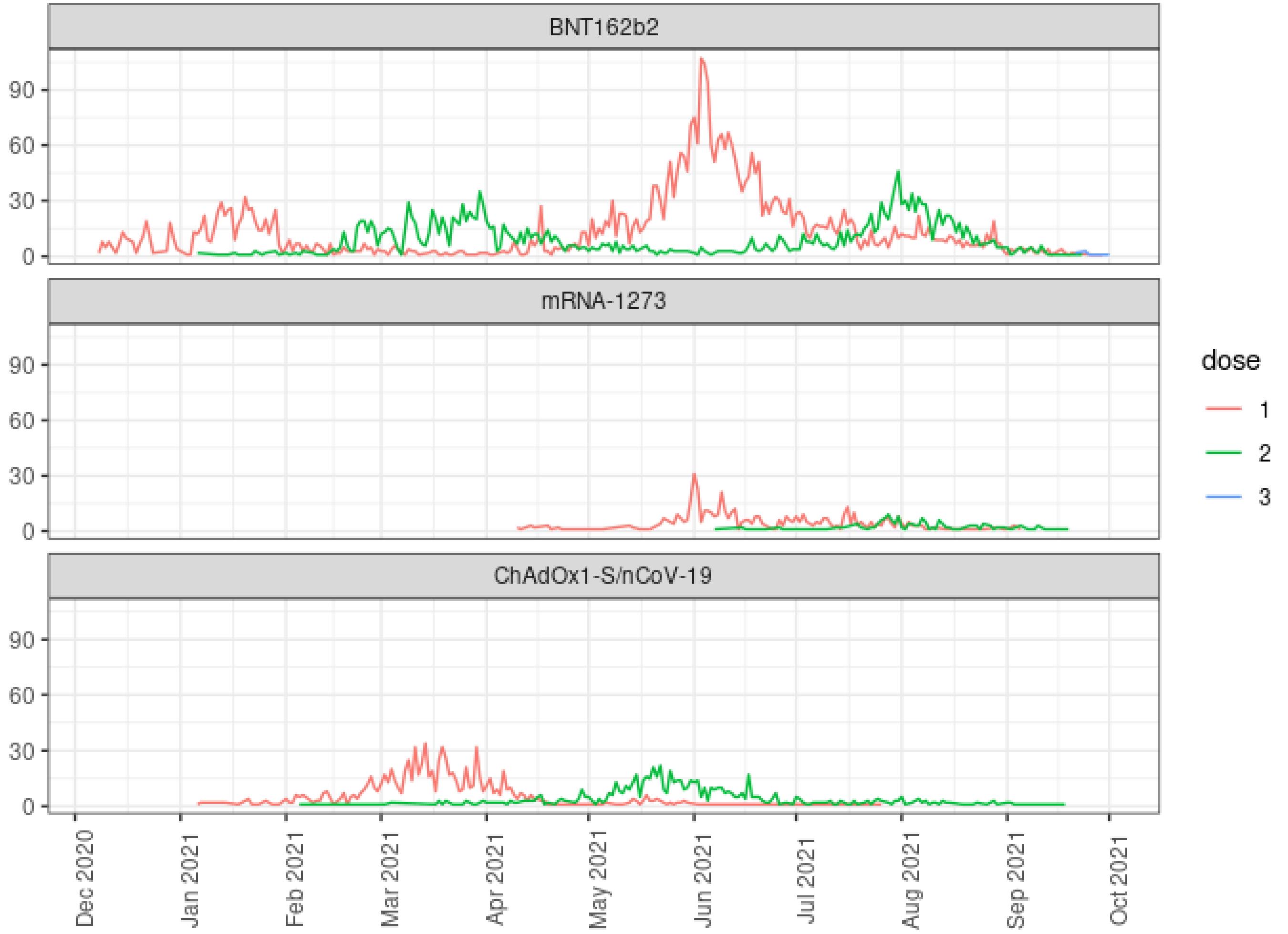

## Supplementary Tables

*Supplementary Table 1: Sensitivity analyses for association between vaccination and major congenital anomalies, calculated using conditional logistic regression models*

| | N pregnancies | N total babies | N babies with any major congenital anomaly | Total prevalence (/1,000 total babies) | OR (95% CI)* | p value* | Adjusted OR (95% CI)** | p value** |
|---|---|---|---|---|---|---|---|---|
| **Sensitivity analysis 1: including babies from pregnancies of any duration** | | | | | | | | |
| **Any major congenital anomaly** | | | | | | | | |
| Unvaccinated | 25318 | 25545 | 466 | 18.2 | 1 | | 1 | |
| Vaccinated | 8407 | 8515 | 153 | 18.0 | 0.98 (0.82-1.18) | 0.87 | 1.01 (0.84-1.22) | 0.90 |
| **Any major non-genetic congenital anomaly** | | | | | | | | |
| Unvaccinated | 25318 | 25545 | 368 | 14.4 | 1 | | 1 | |
| Vaccinated | 8407 | 8515 | 120 | 14.1 | 0.98 (0.79-1.20) | 0.83 | 0.99 (0.80-1.23) | 0.93 |
| **Sensitivity analysis 2: exposure period restricted to conception to 9+6 weeks gestation** | | | | | | | | |
| **Any major congenital anomaly** | | | | | | | | |
| Unvaccinated | 6549 | 6573 | 147 | 22.4 | 1 | | 1 | |
| Vaccinated | 2154 | 2191 | 39 | 17.8 | 0.79 (0.55-1.13) | 0.20 | 0.76 (0.52-1.10) | 0.15 |
| **Any major non-genetic congenital anomaly** | | | | | | | | |
| Unvaccinated | 6549 | 6573 | 123 | 18.7 | 1 | | 1 | |
| Vaccinated | 2154 | 2191 | 29 | 13.2 | 0.70 (0.47-1.06) | 0.09 | 0.66 (0.43-1.02) | 0.06 |

OR=Odds Ratio; CI=Confidence Interval
*Accounting for matching factors: maternal age at conception and gestational week at matching
**In sensitivity analysis 1, additionally adjusted for: maternal deprivation, maternal ethnicity, maternal urban rural status, maternal clinical vulnerability, maternal diabetes, maternal body mass index, and whether the baby was from a singleton or multiple pregnancy. (Maternal smoking status could not be included due to high levels of missing data for pregnancies ending at <12 weeks gestation.)
In sensitivity analysis 2, additionally adjusted as above and for maternal smoking status

*Supplementary Table 2: Key characteristics of vaccinated and control groups included in the subgroup analyses*

| | mRNA vaccines (BNT162b2/mRNA-1273) subgroup analysis | | Viral vector vaccines (ChAdOx1-S/nCoV-19) subgroup analysis | |
|---|---|---|---|---|
| | **Vaccinated cohort** | **Unvaccinated controls** | **Vaccinated cohort** | **Unvaccinated controls** |
| **Key characteristics** | | | | |
| **Number of pregnancies reaching ≥12 weeks gestation** | 5411 | 16352 | 1202 | 3660 |
| **Total babies resulting from these pregnancies** | 5498 | 16494 | 1222 | 3666 |
| **Total live births resulting from these pregnancies** | 5290 | 15843 | 1144 | 3492 |
| **Median maternal age (min-max) [standard deviation]** | 32 (16-46) [5.0] | 32 (16-46) [4.8] | 31 (18-46) [5.4] | 31 (17-47) [5.2] |
| **Maternal deprivation (SIMD quintile)** | | | | |
| 1 (most deprived) | 806 (14.7%) | 3513 (21.3%) | 309 (25.3%) | 822 (22.4%) |
| 2 | 918 (16.7%) | 3192 (19.4%) | 263 (21.5%) | 699 (19.1%) |
| 3 | 1041 (18.9%) | 2987 (18.1%) | 224 (18.3%) | 671 (18.3%) |
| 4 | 1366 (24.8%) | 3655 (22.2%) | 214 (17.5%) | 803 (21.9%) |
| 5 (least deprived) | 1367 (24.9%) | 3133 (19%) | 212 (17.3%) | 668 (18.2%) |
| Unknown | 0 | 14 (0.1%) | 0 | 3 (0.1%) |
| **Maternal ethnicity** | | | | |
| White | 4893 (89%) | 13827 (83.8%) | 1119 (91.6%) | 3095 (84.4%) |
| South Asian | 205 (3.7%) | 610 (3.7%) | 38 (3.1%) | 125 (3.4%) |
| Black/Caribbean /African | 65 (1.2%) | 370 (2.2%) | 20 (1.6%) | 64 (1.7%) |
| Other/mixed ethnicity | 205 (3.7%) | 672 (4.1%) | 24 (2%) | 172 (4.7%) |
| Unknown | 130 (2.4%) | 1015 (6.2%) | 21 (1.7%) | 210 (5.7%) |
| **Maternal urban/rural status** | | | | |
| Large urban areas | 2051 (37.3%) | 6101 (37%) | 421 (34.5%) | 1387 (37.8%) |
| Other urban areas | 1795 (32.6%) | 5850 (35.5%) | 440 (36.0%) | 1221 (33.3%) |
| Accessible small towns | 449 (8.2%) | 1258 (7.6%) | 112 (9.2%) | 289 (7.9%) |
| Remote small towns | 175 (3.2%) | 482 (2.9%) | 51 (4.2%) | 103 (2.8%) |
| Accessible rural areas | 675 (12.3%) | 1915 (11.6%) | 142 (11.6%) | 428 (11.7%) |

| | | | | |
|---|---|---|---|---|
| Remote rural areas | 295 (5.4%) | 725 (4.4%) | 47 (3.8%) | 190 (5.2%) |
| Unknown | 58 (1.1%) | 163 (1%) | 9 (0.7%) | 48 (1.3%) |
| **Maternal clinical vulnerability** | | | | |
| Not vulnerable | 4113 (74.8%) | 12187 (73.9%) | 683 (55.9%) | 2664 (72.7%) |
| Vulnerable | 1341 (24.4%) | 4173 (25.3%) | 477 (39.0%) | 970 (26.5%) |
| Extremely vulnerable | 44 (0.8%) | 134 (0.8%) | 62 (5.1%) | 32 (0.9%) |
| **Maternal diabetes** | | | | |
| No - assumed & confirmed | 5089 (92.6%) | 15136 (91.8%) | 1035 (84.7%) | 3368 (91.9%) |
| Pre-existing diabetes | 40 (0.7%) | 124 (0.8%) | 74 (6.1%) | 31 (0.8%) |
| Gestational Diabetes/onset unknown | 369 (6.7%) | 1234 (7.5%) | 113 (9.2%) | 267 (7.3%) |
| **Maternal smoking status** | | | | |
| Non-smoker | 4088 (74.4%) | 11301 (68.5%) | 791 (64.7%) | 2532 (69.1%) |
| Ex-smoker | 1030 (18.7%) | 3322 (20.1%) | 259 (21.2%) | 705 (19.2%) |
| Smoker | 359 (6.5%) | 1811 (11.0%) | 162 (13.3%) | 413 (11.3%) |
| Unknown | 21 (0.4%) | 60 (0.4%) | 10 (0.8%) | 16 (0.4%) |
| **Maternal body mass index** | | | | |
| Underweight | 85 (1.5%) | 322 (2.0%) | 12 (1.0%) | 84 (2.3%) |
| Healthy weight | 2013 (36.6%) | 5924 (35.9%) | 337 (27.6%) | 1340 (36.6%) |
| Overweight | 1649 (30.0%) | 5094 (30.9%) | 328 (26.8%) | 1101 (30.0%) |
| Obese/severely obese | 1553 (28.2%) | 4597 (27.9%) | 497 (40.7%) | 1002 (27.3%) |
| Unknown | 198 (3.6%) | 557 (3.4%) | 48 (3.9%) | 139 (3.8%) |
| **Baby from singleton or multiple pregnancy** | | | | |
| Singleton | 5324 (96.8%) | 15984 (96.9%) | 1184 (96.9%) | 3563 (97.2%) |
| Multiple | 174 (3.2%) | 510 (3.1%) | 38 (3.1%) | 103 (2.8%) |
| **Exposure (vaccination)** | | | | |
| **Gestation at first vaccination within exposure period*** | | | | |
| Up to six weeks preconception | 1370 (24.9%) | - | 668 (54.7%) | - |
| 2+0-9+6 weeks | 1489 (27.1%) | - | 432 (35.4%) | - |
| 10+0-13+6 weeks | 851 (15.5%) | - | 42 (3.4%) | - |

| | | | | |
|---|---|---|---|---|
| 14+0-19+6 weeks | 1788 (32.5%) | - | 80 (6.5%) | - |
| **Number of vaccinations within exposure period** | | | | |
| 1 | 4044 (73.6%) | - | 827 (67.7%) | - |
| 2+ | 1454 (26.4%) | - | 395 (32.3%) | - |
| **Dose number at first vaccination within exposure period** | | | | |
| Dose 1 | 4607 (83.8%) | - | 898 (73.5%) | - |
| Dose 2 | 880 (16.0%) | - | 324 (26.5%) | - |
| Dose 3 | 11 (0.2%) | - | 0 | - |
| **Outcome (major congenital anomaly)** | | | | |
| **Total N babies with any anomaly** | 110 | 376 | 41 | 91 |
| **Total N live births with any anomaly** | 78 | 270 | 30 | 65 |
| **Total live birth prevalence of any anomaly (/1,000 live births)** | 15 | 17 | 26 | 19 |
| **Total N babies with any non-genetic anomaly** | 84 | 309 | 34 | 66 |
| **Total N live births with any non-genetic anomaly** | 65 | 242 | 27 | 53 |
| **Total live birth prevalence of any non-genetic anomaly (/1,000 live births)** | 12 | 15 | 24 | 15 |
| **N (%) babies with the following types of anomaly [N with non-genetic anomaly]** | | | | |
| Nervous system | 11 (10%) [10] | 35 (9.3%) [35] | 3 (7.3%) [3] | 8 (8.8%) [6] |
| Eye | 2 (1.8%) [2] | 1 (0.3%) [0] | 0 | 0 |
| Ear, face and neck | 2 (1.8%) [2] | 2 (0.5%) [2] | 0 | 0 |
| Congenital heart defects | 30 (27.3%) [26] | 79 (21%) [65] | 11 (26.8%) [9] | 26 (28.6%) [19] |
| Respiratory | 1 (0.9%) [1] | 4 (1.1%) [4] | 0 | 1 (1.1%) [1] |
| Oro-facial clefts | 4 (3.6%) [1] | 15 (4%) [12] | 3 (7.3%) [2] | 1 (1.1%) [1] |
| Digestive system | 9 (8.2%) [9] | 35 (9.3%) [31] | 4 (9.8%) [3] | 10 (11%) [7] |
| Abdominal wall defects | 2 (1.8%) [2] | 4 (1.1%) [4] | 1 (2.4%) [1] | 2 (2.2%) [2] |
| Urinary | 6 (5.5%) [5] | 26 (6.9%) [26] | 5 (12.2%) [2] | 5 (5.5%) [4] |
| Genital | 10 (9.1%) [9] | 31 (8.2%) [31] | 5 (12.2%) [5] | 12 (13.2%) [10] |

| | | | | |
|---|---|---|---|---|
| Limb defect | 16 (14.5%) [15] | 60 (16%) [59] | 5 (12.2%) [5] | 15 (16.5%) [14] |
| Other anomalies/syndromes** | 13 (11.8%) [6] | 22 (5.9%) [14] | 5 (12.2%) [3] | 8 (8.8%) [4] |
| Chromosomal | 14 (11.8%) [0] | 59 (15.7%) [0] | 5 (12.2%) [0] | 21 (23.1%) [0] |

SIMD=Scottish Index of Multiple Deprivation

*Between 6 weeks preconception and up to the earliest of: (1) end of pregnancy or (2) 19 weeks 6 days gestation.

**The "Other anomalies/syndromes group" includes a disparate range of conditions including genetic syndromes and microdeletions, skeletal dysplasias, and recognised teratogenic syndromes and associations. No unusual pattern in the distribution of these other anomalies was seen in either of the vaccinated groups.

*Supplementary Table 3: Subgroup analysis of association between vaccination and major congenital anomalies by vaccine group, calculated using conditional logistic regression models*

| | N pregnancies | N total babies | N babies with any major congenital anomaly | Total prevalence (/1,000 total babies) | OR (95% CI)* | p value* | Adjusted OR (95% CI)** | p value** |
|---|---|---|---|---|---|---|---|---|
| **mRNA vaccines (BNT162b2/mRNA-1273) subgroup analysis** | | | | | | | | |
| **Any major congenital anomaly** | | | | | | | | |
| Unvaccinated | 16352 | 16494 | 376 | 22.8 | 1 | | 1 | |
| Vaccinated | 5411 | 5498 | 110 | 20.0 | 0.87 (0.70-1.08) | 0.22 | 0.92 (0.73-1.14) | 0.44 |
| **Any major non-genetic congenital anomaly** | | | | | | | | |
| Unvaccinated | 16352 | 16494 | 309 | 18.7 | 1 | | 1 | |
| Vaccinated | 5411 | 5498 | 84 | 15.3 | 0.81 (0.64-1.04) | 0.09 | 0.85 (0.66-1.10) | 0.22 |
| **Viral vector vaccines (ChAdOx1-S/nCoV-19) subgroup analysis** | | | | | | | | |
| **Any major congenital anomaly** | | | | | | | | |
| Unvaccinated | 3660 | 3666 | 91 | 24.8 | 1 | | 1 | |
| Vaccinated | 1202 | 1222 | 41 | 33.6 | 1.35 (0.94-1.96) | 0.11 | 1.35 (0.90-2.03) | 0.15 |
| **Any major non-genetic congenital anomaly** | | | | | | | | |
| Unvaccinated | 3660 | 3666 | 66 | 18.0 | 1 | | 1 | |
| Vaccinated | 1202 | 1222 | 34 | 27.8 | 1.55 (1.02-2.35) | 0.04 | 1.56 (0.97-2.51) | 0.06 |

OR=Odds Ratio; CI=Confidence Interval

*Accounting for matching factors: maternal age at conception and gestational week at matching

**Additional adjusted for: maternal deprivation, maternal ethnicity, maternal urban rural status, maternal clinical vulnerability, maternal diabetes, maternal body mass index, maternal smoking status, and whether the baby was from a singleton or multiple pregnancy

*Supplementary Table 4: Sensitivity analyses for association between SARS-CoV-2 infection and major congenital anomalies, calculated using conditional logistic regression models*

| | N pregnancies | N total babies | N babies with any major congenital anomaly | Total prevalence (/1,000 total babies) | OR (95% CI)* | p value* | Adjusted OR (95% CI)** | p value** |
|---|---|---|---|---|---|---|---|---|
| **Sensitivity analysis 1: including babies from pregnancies of any duration** | | | | | | | | |
| **Any major congenital anomaly** | | | | | | | | |
| Uninfected | 5472 | 5478 | 90 | 16.4 | 1 | | 1 | |
| Infected | 1803 | 1826 | 32 | 17.5 | 1.07 (0.71-1.60) | 0.75 | 0.93 (0.60-1.45) | 0.74 |
| **Any major non-genetic congenital anomaly** | | | | | | | | |
| Uninfected | 5472 | 5478 | 77 | 14.1 | 1 | | 1 | |
| Infected | 1803 | 1826 | 26 | 14.2 | 1.01 (0.65-1.59) | 0.95 | 0.93 (0.58-1.52) | 0.79 |
| **Sensitivity analysis 2: exposure period restricted to conception to 9+6 weeks gestation** | | | | | | | | |
| **Any major congenital anomaly** | | | | | | | | |
| Uninfected | 1548 | 1548 | 30 | 19.4 | 1 | | 1 | |
| Infected | 508 | 516 | 12 | 23.3 | 1.20 (0.61-2.37) | 0.59 | 1.14 (0.57-2.29) | 0.72 |
| **Any major non-genetic congenital anomaly** | | | | | | | | |
| Uninfected | 1548 | 1548 | 24 | 15.5 | 1 | | 1 | |
| Infected | 508 | 516 | 10 | 19.4 | 1.26 (0.60-2.65) | 0.55 | 1.05 (0.47-2.34) | 0.91 |

OR=Odds Ratio; CI=Confidence Interval

*Accounting for matching factors: maternal age at conception, season of conception and gestational week at matching

**In sensitivity analysis 1, additionally adjusted for: maternal deprivation, maternal ethnicity, maternal urban rural status, maternal clinical vulnerability, maternal diabetes, maternal body mass index, and whether the baby was from a singleton or multiple pregnancy. (Maternal smoking status could not be included due to high levels of missing data for pregnancies ending at <12 weeks gestation.)

In sensitivity analysis 2, additionally adjusted for: maternal urban rural status, maternal clinical vulnerability, maternal diabetes, and maternal BMI. (Restricted range of covariates used due to the small numbers involved.)
