# Supplementary material for: A population-based matched cohort study of major congenital anomalies following COVID-19 vaccination and SARS-CoV-2 infection
Source: Nat Commun. 2023 Jan 6;14:107. doi: 10.1038/s41467-022-35771-8 (PMC9821346; doi:10.1038/s41467-022-35771-8)
Supplement: Supplementary file 3 — Reporting Summary [file 41467_2022_35771_MOESM3_ESM.pdf]

nature portfolio

Corresponding author(s): Rachael Wood

Last updated by author(s): Dec 13, 2022

Reporting Summary

Nature Portfolio wishes to improve the reproducibility of the work that we publish. This form provides structure for consistency and transparency in reporting. For further information on Nature Portfolio policies, see our Editorial Policies and the Editorial Policy Checklist.

Statistics

For all statistical analyses, confirm that the following items are present in the figure legend, table legend, main text, or Methods section.

n/a	Confirmed	
☐	☒	The exact sample size (*n*) for each experimental group/condition, given as a discrete number and unit of measurement
☐	☒	A statement on whether measurements were taken from distinct samples or whether the same sample was measured repeatedly
☐	☒	The statistical test(s) used AND whether they are one- or two-sided<br>*Only common tests should be described solely by name; describe more complex techniques in the Methods section.*
☐	☒	A description of all covariates tested
☐	☒	A description of any assumptions or corrections, such as tests of normality and adjustment for multiple comparisons
☐	☒	A full description of the statistical parameters including central tendency (e.g. means) or other basic estimates (e.g. regression coefficient) AND variation (e.g. standard deviation) or associated estimates of uncertainty (e.g. confidence intervals)
☐	☒	For null hypothesis testing, the test statistic (e.g. *F*, *t*, *r*) with confidence intervals, effect sizes, degrees of freedom and *P* value noted<br>*Give P values as exact values whenever suitable.*
☒	☐	For Bayesian analysis, information on the choice of priors and Markov chain Monte Carlo settings
☒	☐	For hierarchical and complex designs, identification of the appropriate level for tests and full reporting of outcomes
☒	☐	Estimates of effect sizes (e.g. Cohen's *d*, Pearson's *r*), indicating how they were calculated

Our web collection on statistics for biologists contains articles on many of the points above.

Software and code

Policy information about availability of computer code

Data collection	R version 3.6.1
Data analysis	R version 3.6.1 with code available at: https://github.com/Public-Health-Scotland/COPS-public

For manuscripts utilizing custom algorithms or software that are central to the research but not yet described in published literature, software must be made available to editors and reviewers. We strongly encourage code deposition in a community repository (e.g. GitHub). See the Nature Portfolio guidelines for submitting code & software for further information.

Data

Policy information about availability of data

All manuscripts must include a data availability statement. This statement should provide the following information, where applicable:

- Accession codes, unique identifiers, or web links for publicly available datasets
- A description of any restrictions on data availability
- For clinical datasets or third party data, please ensure that the statement adheres to our policy

Aggregate data files on COVID-19 vaccinations and SARS-CoV-2 infections among pregnant women are available here: https://www.opendata.nhs.scot/organization/health_protection. Meta-data are available here: https://github.com/Public-Health-Scotland/COPS-public. Patient-level data underlying this article cannot be shared publicly due to data protection and confidentiality requirements. Public Health Scotland is the data holder for the data used in this study. Data can be made

available to approved researchers for analysis after securing relevant permissions from the data holders via the Public Benefit and Privacy Panel. Enquiries regarding data availability should be directed to phs.edris@phs.scot.

Human research participants

Policy information about studies involving human research participants and Sex and Gender in Research.

Reporting on sex and gender	We refer to women/mothers throughout the paper when discussing the exposure in pregnancy and maternal socio-demographic and clinical characteristics. No data are presented on sex of babies as this is not relevant to our study question.
Population characteristics	Fetuses/babies ending in early pregnancy loss, termination of pregnancy, live or stillbirth. Firstly, we selected all babies exposed to maternal COVID-19 vaccination (but not SARS-CoV-2 infection) between six weeks preconception and 19 weeks and 6 days gestational as well as 3 control babies for each vaccinated baby who were not exposed to either maternal COVID-19 vaccination or SARS-CoV-2 infection in the same pregnancy risk period matched on maternal age at conception and gestational age at time of vaccination/matching. Secondly, we selected all babies exposed to SARS-CoV-2 infection (but not COVID-19 vaccination) between six weeks preconception and 19 weeks and 6 days gestational as well as 3 control babies for each vaccinated baby who were not exposed to either maternal COVID-19 vaccination or SARS-CoV-2 infection in the same pregnancy risk period matched on maternal age at conception, season of conception and gestational age at time of infection/matching. We provide detailed descriptive information on these cohorts in Table 1 and Table 4 in the paper.
Recruitment	National, prospective dynamic cohort of routinely collected healthcare data.
Ethics oversight	The National Research Ethics Service Committee, South East Scotland 02 provided ethical approval for COPS (REC 12/SS/0201: SA 2). The Public Benefit and Privacy Panel for Health and Social Care provided information governance approval (2021-0116).

Note that full information on the approval of the study protocol must also be provided in the manuscript.

Field-specific reporting

Please select the one below that is the best fit for your research. If you are not sure, read the appropriate sections before making your selection.

☒ Life sciences ☐ Behavioural & social sciences ☐ Ecological, evolutionary & environmental sciences

For a reference copy of the document with all sections, see nature.com/documents/nr-reporting-summary-flat.pdf

Life sciences study design

All studies must disclose on these points even when the disclosure is negative.

Sample size	Sample size calculations were conducted and are presented in the protocol. We ultimately included all eligible babies in our exposed groups based on this national level dataset, with appropriate numbers of unexposed controls selected.
Data exclusions	Data were excluded if there were pre-specified unfeasible values, as described in the data dictionary available from: https://github.com/Public-Health-Scotland/COPS-public.
Replication	We undertook two sensitivity analyses varying our exposure window and the inclusion criteria for babies for analysis, neither of which changed our conclusions, suggesting that our results were robust to our study population and exposure window. More generally, all code was double checked by a second analyst to ensure it was correct.
Randomization	This was an observational study so we did not conduct randomization. We controlled for confounders by matching our exposed groups of babies to unexposed groups of babies (1:3 matching) by key confounders (e.g., maternal at at conception), with other confounders adjusted for in the conditional logistic regression model (e.g, area-level deprivation and clinical vulnerability).
Blinding	This study relied on national level data drawn from routine health records, with all eligible babies were included in the dataset and analysis. Analysts running the main models were not blinded to the exposure status of babies in the analyses. To minimize the potential for any bias introduced for lack of blinding, we prepared a detail protocol and analysis plan that was followed throughout the analysis.

Reporting for specific materials, systems and methods

We require information from authors about some types of materials, experimental systems and methods used in many studies. Here, indicate whether each material, system or method listed is relevant to your study. If you are not sure if a list item applies to your research, read the appropriate section before selecting a response.

Materials & experimental systems

n/a	Involved in the study
☒	☐ Antibodies
☒	☐ Eukaryotic cell lines
☒	☐ Palaeontology and archaeology
☒	☐ Animals and other organisms
☒	☐ Clinical data
☒	☐ Dual use research of concern

Methods

n/a	Involved in the study
☒	☐ ChIP-seq
☒	☐ Flow cytometry
☒	☐ MRI-based neuroimaging
